# Training load responses modelling and model generalisation in elite sports

**DOI:** 10.1038/s41598-022-05392-8

**Published:** 2022-01-28

**Authors:** Frank Imbach, Stephane Perrey, Romain Chailan, Thibaut Meline, Robin Candau

**Affiliations:** 1 Seenovate, Montpellier, France; 2 EuroMov Digital Health in Motion, Univ Montpellier, IMT Mines Ales, Montpellier, France; 3 DMeM, INRAe, Univ Montpellier, Montpellier, France; 4 Fédération Française des Sports de Glace, Paris, France

**Keywords:** Computational models, Machine learning, Systems biology, Computer science, Predictive markers

## Abstract

This study aims to provide a transferable methodology in the context of sport performance modelling, with a special focus to the generalisation of models. Data were collected from seven elite Short track speed skaters over a three months training period. In order to account for training load accumulation over sessions, cumulative responses to training were modelled by impulse, serial and bi-exponential responses functions. The variable dose-response (DR) model was compared to elastic net (ENET), principal component regression (PCR) and random forest (RF) models, while using cross-validation within a time-series framework. ENET, PCR and RF models were fitted either individually ($M_{I}$) or on the whole group of athletes ($M_{G}$). Root mean square error criterion was used to assess performances of models. ENET and PCR models provided a significant greater generalisation ability than the DR model ($p = 0.018$, $p < 0.001$, $p = 0.004$ and $p < 0.001$ for $ENET_{I}$, $ENET_{G}$, $PCR_{I}$ and $PCR_{G}$, respectively). Only $ENET_{G}$ and $RF_{G}$ were significantly more accurate in prediction than DR ($p < 0.001$ and $p < 0.012$). In conclusion, ENET achieved greater generalisation and predictive accuracy performances. Thus, building and evaluating models within a generalisation enhancing procedure is a prerequisite for any predictive modelling.

## Introduction

The relationship between training load and performance in sports has been studied since decades. A key point of the performance optimisation is the training prescription delivered by coaches, physical trainers or the athlete himself. Such a programming involves both various modalities of exercise (i.e. the type of training regarding to the physical quality required to perform) and adjusted training load. Training load is usually dissociated into (i) an external load defined by the work completed by the athlete, independently of his internal characteristics^1^ and (ii) an internal load corresponding to the psycho-physiological stresses imposed on the athlete in response to the external load^2^.

Models of training load responses emerged with the impulse response model promoted by Banister et al.^3^ in order to describe human adaptations to training loads. Afterwards, a simplified version of the original model built on a two-way antagonistic first order transfer function (fitness and fatigue components, so called Fitness–Fatigue model) has showed a large interest to describe the training process^4–8^. However, several limitations regarding to the model stability, parameter interpretability, ill-conditioning and predictive accuracy were reported^9,10^. Such models are considered as time-varying linear models according to their component structure^11^ and therefore, may require a sufficient number of observations (i.e. performances) to correctly estimate relationships between training load and performance^9,12^. To overcome some of the limits, refinements of the former impulse response model were proposed by using a recursive algorithm in order to estimate parameters according to each model input (i.e. the training load)^11^ and by introducing variations in the fatigue response to a single training bout^13^. Further adaptations to the Fitness–Fatigue model were also developed with the aim of improving both goodness-of-fit and prediction accuracy^14,15^. Nonetheless, impulse response models sought to mitigate the underpinning physiological processes involved by exercise into a small number of entities for predicting training effects in both endurance (running, cycling, skiing and swimming)^6,11,16–21^ and more complex (hammer throw, gymnastic and judo)^8,22,23^ activities. This simplistic approach might prevent from catching the appropriate relationship between training and performance, and finally impair accuracy of predictions^24^. Moreover, with the exception of the one from Matabuena et al.^15^, these models assume that the training effect is maximal by the end of the training session. This assumption is reasonable only for the negative component of the model (i.e. “Fatigue”), where its maximal value is taken immediately after the session. Regarding to the positive effects induced by training (i.e. “Fitness”), such a response is quite questionable since physiological adaptations are continuing from the end of the exercise session. For instance, skeletal muscle adaptations to training described by increases in muscle mass, fiber shortening velocity and myosin ATPase activity modifications are known to be progressive (i.e. short to long term after-effects) rather than instantaneous^25–27^. Consequently, serial and bi-exponential functions were proposed to counteract these limitations and better describe training adaptations through exponential growth and decay functions, according to physiological responses in rats^28^.

A more statistical approach was used to investigate the effects of training load on performance by using principal component analysis and linear mixed models on different time frames^12^. Such models infer parameters from all available data (i.e. combining subjects instead of by-subject model) but allow parameters to vary regarding the heterogeneity between athletes. The model being multivariate, the multi-faceted nature of the performance could be conserved by including psychological, nutritional and technical information as predictors^12,16,18^. However, authors did not consider the cumulative facet of daily training loads, where exponential and decay cumulative functions such as proposed by Candau et al.^17^ may be suitable for performance modelling.

Alternatives from computer sciences field were also used to clarify the training load - performance relationship in a predictive aim. Most notably, machine learning approaches are usually focused on the generalisation of models (i.e. how accurately a model is able to predict outcome values for previously unseen data). Various approaches tend to maximise such a criterion. For instance, one can perform cross-validation (CV) procedures, where data are separated into training sets for parameters estimation and testing sets for prediction^29^. Such a procedure fosters the determination of optimal models, relatively to the family of models considered and regarding to their ability for generalisation. In the same time, CV procedures allow to diagnose under- and over-fitting of the model. Underfitting commonly describes an inflexible model unable of capturing noteworthy regularities in a set of exemplary observations^30^. In contrast, overfitting represents an over-trained model, which tends to memorise each particular observation thus leading to high error rates when predicting on unknown data^31^. While aforementioned studies aimed to describe the training load - performance relationships by estimating model parameters and by testing the model on a single data set, generalisation of models cannot be ensured. This challenges their usefulness in a predictive application. On the other hand, modelling methodologies using CV procedures are the standard in a predictive aim rather than only being descriptive. To our knowledge, only a few recent studies modelled performances with Fitness–Fatigue models using a CV procedure^10,32,33^ and one separated data into two equals training and testing data sets respectively^34^. Ludwig et al.^10^ reported that optimising all parameters including the offset term makes the model prone to overfitting. Consequently, interpretations drawn from predictions as well as model parameters may be incorrect.

The physiological adaptations involved by exercise being complex, some authors investigated the relationship between training and performance by using Artificial Neural Networks (ANN), non-linear machine learning models^35,36^. Despite low prediction errors reported (e.g. 0.05 seconds error over a 200m swimming performance^35^), the methodological consideration in their study mostly influenced by a small sample size and the “black-box” nature of ANN question their use in sport performance modelling^9,37^. Computer sciences offer plenty of machine learning models although being often summarised in ANN for athletic performance prediction. Considering labelled athletic performances, powerful algorithms from supervised learning could be alternatively considered for solving athletic performance modelling issues, either through a regression or a classification formulation of the problem. To cite a few, non-linear approaches such as Random Forest (RF) models account for the non-linear relationships between a target and a *large* set of predictors^38^ for making predictions. In a different way, linear models such as regularised linear regressions^39,40^ also proved their efficiency in high dimensionality and multicollinearity contexts. On this basis, both could be profitable for sport performance modelling purposes.

To date, not any model family (i.e. impulse response and physiological based, statistical and machine learning models) seems to be preferred for athletic performance prediction based on a data set, mainly due to a lack of evidence and confidence in training effect modelling and performance prediction accuracy. In addition, because generalisation ability is not systemically appraised, practical and physiological interpretations drawn from some models may be incorrect and at least should be taken with caution.

In order to elucidate the relationships between training loads and athletic performance in a predictive application, we hypothesised that following a model selection, regularisation and dimension reduction methods would lead to a greater model generalisation capability than former impulse response models.

Aiming to prescribe an optimal training programming, sport practitioners need to understand the physiological effects involved by each training session and its after-effects on athletic performance. Hence, this study aimed to provide a robust and transferable methodology relying on model generalisation in a context of sport performance modelling. We collected data from elite Short-track speed skaters, part of the National French team. To date, only a few studies have investigated relationships between training and performances in this sport^41–43^. From linear and non-linear modelling approaches, Knobbe et al.^42^ provided an interesting methodology around aggregation methods for delivering key and actionable features of training components. The authors investigated individual patterns that represent adaptations to training and that might provide insightful information for coaches, involved in training programming tasks. On another note, Meline et al.^43^ examined the dose-response relationship between training and performance through simulations of overloading and a few tapering strategies. The dose-response model from Busso^13^ appeared to be a valuable model for evaluating taper strategies and their potential effects on skating performance. However, a contribution mostly based on the model generalisation principle seems to be of interest by reinforcing the knowledge of athletic performance modelling in elite sports.

After having constructed an appropriate data set, we considered the variable dose-response model (DR)^13^ as a baseline regression framework and compared it to three models: a principal component regression (PCR), an Elastic net (ENET) regularised regression and a RF regression model. These models allow:

1. To present and discuss the help of regularisation and dimension reduction methods in regards of the generalisation concept.
2. To model athletic performances using robust models to the high dimensionality and multicollinearity and to investigate the key factors of the short-track speed skating performance.

## Materials and methods

### Participants

Seven national elite Short-track speed skaters (mean age 22.7 ± 3.4 years old; 3 males, body mass of 71.4 ± 9.4 kg, and 4 females, body mass of 55.9 ± 3.9 kg) voluntary participated to the study. Each athletes experienced the 2018 Olympic Winter Games in PyeongChang, South Korea ($n = 2$) or were preparing the Olympics Games of Pekin, China ($n = 7$). The whole team was trained by the same coach, responsible for training programming and data collection. Mean weekly volume of training was 16.6 ± 2.5 hours. Data were collected over a three months training period without any competition, interrupted by a two weeks break and beginning one month after resuming training for a new season. Participants were fully informed about data collection and written consent was obtained from them. The study was performed in agreement with the standards set by the declaration of Helsinki (2013) involving human subjects. The protocol was reviewed and approved by the local research Ethics Committee (EuroMov, University of Montpellier, France). The present retrospective study relied on the collected data without causing any changes in the training programming of athletes.

### Data set

#### Dependent variable: performance

Participants performed each week standing start time trials ($distance = 166.68 \, \text {meters}$ equal 1.5 lap) after a standardised warm-up. At the finish line, timing gates system (Brower timing system, USA) recorded individual time trial performance in order to ensure a high standard of validity and reliability between measures^44,45^. A total of $n=248$ performances were recorded for an average of $35.4 \pm 2.23$ individual performances. The performance test being a gold standard for the assessment of acceleration ability^46^, athletes were all familiar with it prior to the study.

In the sequel, let ${\mathscr {Y}} \subset {\mathbb {R}}$ be the domain of definition of such a performance and $Y \in {\mathscr {Y}}$ the continuous random variable. In this context, each observation $y_j \in Y$ can be referenced by both its athlete *i* and its day of realisation *t* as $y_{i,t}$.

#### Independent variables

**Figure 1:**
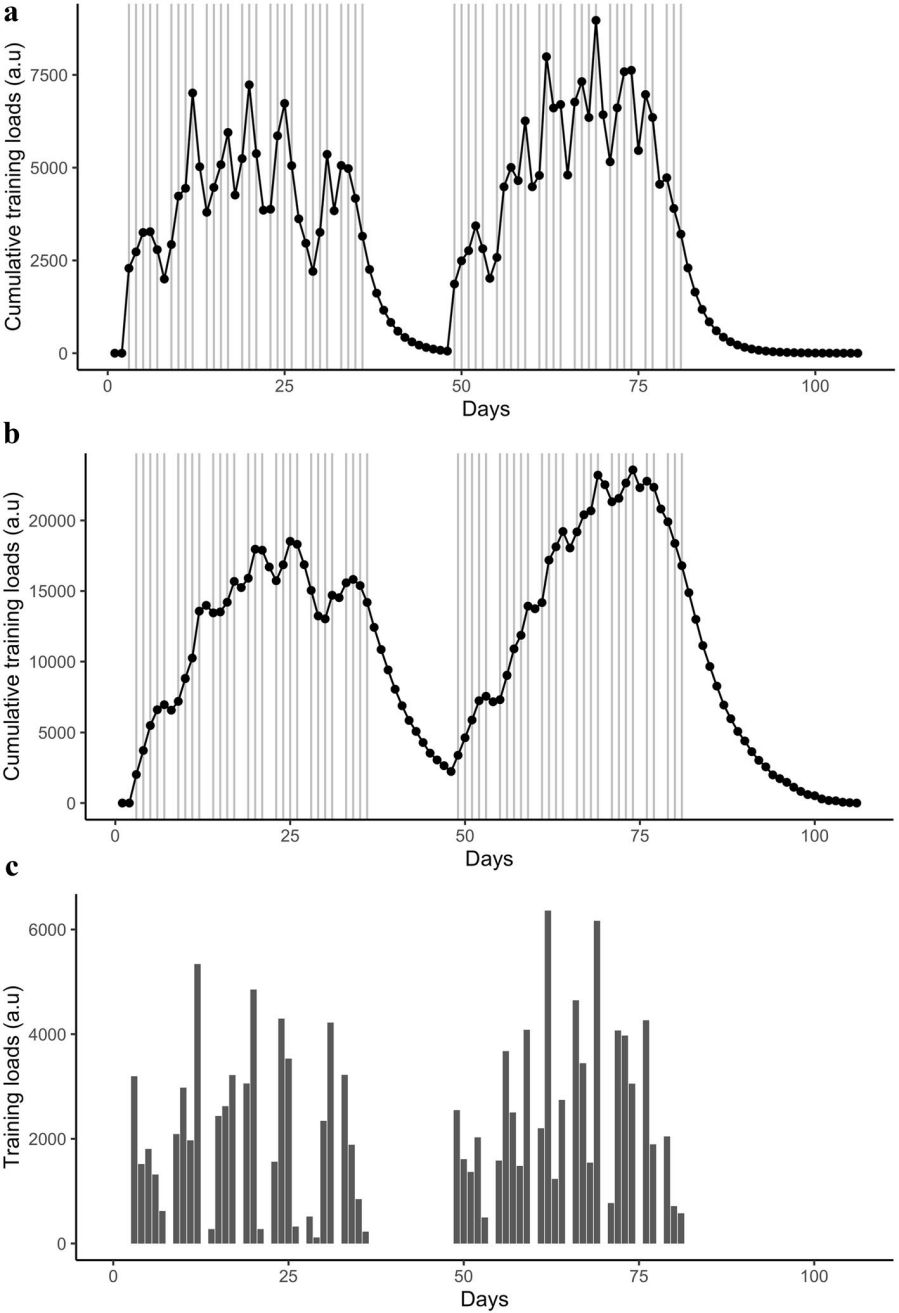
Cumulative daily training loads of a representative athlete following (**a**) the impulse response function ($X_2$, Eq. 1) and (**b**) the serial bi-exponential response function ($X_3$, Eq. 2). (**c**) illustrates the raw daily training loads $X_1$, expressed by w(t). In (**a**) and (**b**), dots represent daily values of the cumulative training load and vertical solid lines indicate occurrence of training sessions. Values are represented in arbitrary units (a.u).

Independent variables stem from various sources, which are summarised in Table 1. In the sequel, let ${\mathscr {X}} \subset {\mathbb {R}}^d \, \text {with} \, d \in {\mathbb {N}}$ be the domain of definition of the random variable $X = [X_1, \ldots , X_d] \in {\mathscr {X}}$. The variable *X* is thus defined as a vector composed of the independent variables detailed hereafter. First, $\{X_1\}$ refers to the raw training loads (TL, Fig. 1c), calculated from on-ice and off-ice training sessions (see details on Supplementary material Appendix 1). Then, $\{X_2,X_3\}$ represent two aggregations of daily TL. Those aggregations come from the daily training loads *w*(*t*)—also known here as $X_1$—convoluted to two transfer functions adapted from Philippe et al.^28^, which are denoted $g_{\text {imp}}(t)$ and $g_{\text {ser}}(t)$.

The associated impulse response $G_{\text {imp}}(t)$ reflects the acute response to exercise (e.g. fatigue). It is defined as

1 $$\begin{aligned} G_{\text {imp}}(t) = e^{\frac{-t}{\tau _I}} \, , \end{aligned}$$

where $\tau _I$ is a short time constant equals to 3 days in this study (Fig. 1a). Respectively, the response $G_{\text {ser}}(t)$ describes a serial and bi-exponential function reflecting training adaptations over time. It is defined as

2 $$\begin{aligned} G_{\text {ser}}(t) = \big (1 - e^{\frac{-t}{\tau _G}}\big ) \, U \, + \, e^ {\frac{-(t-TD)}{\tau _D}} \, \mid U-1 \mid \, , \quad \text {with} \quad U= {\left\{ \begin{array}{ll} 1 &{}\quad \text {if} \quad t < TD\\ 0 &{}\quad \text {otherwise.} \end{array}\right. } \end{aligned}$$

The time delay for the decay phase to begin only after the growth phase is given by the constant *TD*. Here, $TD = 4 \tau _G$. Both $\tau _G$ and $\tau _D$ are the time constants of respectively the growth phase and the decline phase with $\tau _G = 1 \, \text {day}$ and $\tau _D = 7 \, \text {days}$ (Fig. 1b). Note that the time constants $\tau _I$, $\tau _G$, $\tau _D$ were averaged and based on empirical knowledge and previous findings^13^. Hence, for a given athlete,

$$\begin{aligned} X_2(t)&= \left( w * g_{\text {imp}} \right) (t) = \sum _{l=1}^{t} w(l) \left( e^{\frac{-(t-l)}{\tau _I}}\right) \, , \quad \text {and} \\ X_3(t)&= \left( w * g_{\text {ser}} \right) (t) = \sum _{l=1}^{t} w(l) \left( \big (1 - e^{\frac{-(t-l)}{\tau _G}}\big ) \, U \, + \, e^ {\frac{-(t-TD-l)}{\tau _D}} \, \mid U-1 \mid \right) \, , \quad \text {with} \quad U= {\left\{ \begin{array}{ll} 1 &{}\quad \text {if} \quad t < TD\\ 0 &{}\quad \text {otherwise.} \end{array}\right. } \end{aligned}$$

Note that the symbol $*$ denotes the convolution product.

Similarly, some characteristics components of each session were aggregated. This encompasses Rate of Perceived Exertion (RPE) $\{X_4, X_5\}$, averaged power $\{X_6, X_7\}$, maximal power output $\{X_{8}, X_{9}\}$, relative intensity $\{X_{10}, X_{11}\}$, session duration $\{X_{12}, X_{13}\}$ and session density $\{X_{14}, X_{15}\}$. Also, for each session ice quality $\{X_{16}\}$ and rest between two consecutive sessions $\{X_{17}\}$ were considered. Since some models may benefit from time through autocorrelated performances $y_{i,t}$, the preceding performance $y_{i,t-k}$ with $k=1$ was included as predictor, denoted $\{X_{18}\}$. Finally, athlete $\{X_{19}\}$ was considered excepted for individually built models.

Applied to the observed data of this study a data set of $n =248$ observations of performances associated with 19 independent variables was obtained (see Table 1). To formalise, allowing that $X \times Y \sim f$ with *f* a function of density, the built data set is a sample $S = \{(x_j, y_j)\}_{j \le n} \sim f^n$.

**Table 1:** Summary of independent variables.

Independent variables	${\text {X}_\text {i}}$	Description	Aggregation
Raw training load	$X_1$	Daily training load computed from $TL_{ice}$, $TL_{RT}$, $TL_{aer}$, $TL_{RS}$, $TL_{act}$ (see Supplementary material Appendix 1)	Daily recorded
Cumulative Training load	$X_2$, $X_3$	Daily computed from $X_1$ values	Impulse and serial cumulative responses
Rate of Perceived Exertion (RPE)	$X_4$, $X_5$	Borg category ratio (CR) 0–10 scale	Impulse and serial cumulative responses
Averaged power	$X_6$, $X_7$	On-ice sessions	Impulse and serial cumulative responses
Maximal power	$X_8$, $X_9$	On-ice sessions	Impulse and serial cumulative responses
Relative intensity	$X_{10}$, $X_{11}$	On-ice sessions (see Supplementary material Appendix 1, Eq. S1)	Impulse and serial cumulative responses
Session duration	$X_{12}$, $X_{13}$	All sessions, overall session duration	Impulse and serial cumulative responses
Session density	$X_{14}$, $X_{15}$	All sessions, effective work only	Impulse and serial cumulative responses
Ice quality	$X_{16}$	Subjective information quoted on a Borg 0–10 CR scale	Recorded the day of performance
Rest	$X_{17}$	Rest between two consecutive sessions (days)	Sum of rest days preceding the performance
Past performance	$X_{18}$	Significantly correlated past $\text {performance}_{t-k}$ with $\text {performance}_t$	Performance at $\text {day}_{t-k}$
Athlete	$X_{19}$	Athlete’s id	

The two aggregation methods (impulse ans serial cumulative responses) are defined in Eqs. (1) and (2).

### Modelling methodology

Formally, the goal is to find a function $h : X \rightarrow Y$ which minimises the generalisation error

$$\begin{aligned} R(h) = {\mathbb {P}}(h(X) \ne Y ) = {\mathbb {E}}[ {\mathbbm{1}} [h(X) \ne Y]]. \end{aligned}$$

In practice the minimisation of *R* is unreachable. Instead, we get a sample set $S={(x_i, y_i)}_{i \le n} \in X \times Y$ and note the empirical error as

$$\begin{aligned} Re(h) = \frac{1}{n} \sum _{i}^n [ {\mathbbm{1}} [ h(x_i) \ne y_i]]. \end{aligned}$$

The objective becomes to find the best estimate ${{\hat{h}}} = {{\,\mathrm{\mathrm{argmin}}\,}}_{h\in {\mathscr {H}}} Re(h)$ with ${\mathscr {H}}$ the class of function that we accept to consider.

Here, four family of models are evaluated in this context. With the exception of the DR, all models were individually and collectively computed ($h_I$ and $h_G$, respectively).

#### Reference: variable dose-response

The time-varying linear mathematical model developed by Busso^13^ was considered as the model of reference. Formally and according to the previously introduced notation, this model is a function $h^{\text{( }busso)} : X_1 \rightarrow Y$. It describes the training effects on performance over time, *y*(*t*), from the raw training loads $X_{1}$. TL are convoluted to a set of transfer functions $g_\text {apt}(t)$ and $g_\text {fat}(t)$, relating respectively to the aptitude and to the fatigue impulse responses as

$$\begin{aligned} g_\text {apt}(t)&= e^{\frac{-t}{\tau _1}} \\ g_\text {fat}(t)&= e^{\frac{-t}{\tau _2}} , \end{aligned}$$

with $\tau _1$ and $\tau _2$ two time constants. Combined with the basic level of performance $y^*$ of the athlete, the modelled performance is

$$\begin{aligned} {{\hat{y}}}^\text {(busso)}(t) = y^* + k_1 (w*g_\text {apt})(t) - ((k_2 w)*g_\text {fat})(t) \, , \end{aligned}$$

with $k_1$ and $k_2(t)$ being gain terms. The later is related to the training doses by a second convolution to the transfer function

$$\begin{aligned} g_\text {fat'}(t) = e^{\frac{-t}{\tau _3}} \, , \end{aligned}$$

with $\tau 3$ a time constant. Since is defined as $k_2(t) = k_3 (w*g_{\text {fat'}})(t)$ where $k_3$ is a gain term, one may note that $k_2(t)$ increases proportionally to the training load and decay decreases exponentially from this new value. From discrete convolutions, the modelled performance can be rewritten as

$$\begin{aligned} {{\hat{y}}}^\text {(busso)}(t) = y^{*} + k_1 \, \sum _{l=1}^{t-1} w(l) e^{\frac{-(t-l)}{\tau _1}} - \sum _{l=1}^{t-1} k_{2}(l) w(l) e^{\frac{-(t-l)}{\tau _2}}, \end{aligned}$$

with $$k_{2}(l) = k_{3} \sum _{m=1}^{l} w(m) e^{\frac{-(l-m)}{\tau _3}}.$$

The five parameters of the model (i.e. $k_1$, $k_3$, $\tau _{1}$, $\tau _{2}$ and $\tau _{3}$) are fitted by minimizing the residual sum of squares (RSS) between modelled and observed performances, such as

$$\begin{aligned} RSS = \sum _{t=1}^{T} ( {{\hat{y}}}^\text {(busso)}(t) - y(t) )^2 \, , \end{aligned}$$

where $t \in T$ being the day in which the performance is measured. A non-linear minimisation was employed according to a Newton-type algorithm^47^.

Unlike this model of reference, the next presented models take benefit from the augmented data space $X^* = X \, \backslash \, X_1$.

#### Regularisation procedures

##### Elastic net

 In highly dimensional contexts, multivariate linear regressions may lead to unsteady models by being excessively sensitive to the expanded space of solutions. To tackle this issue, cost functions penalising some parameters on account of correlated variables exist. On one side, Ridge penalisation reduces the space of possible functions by assigning a constraint to the parameters, thus minimising their amplitude to almost null values. On the other side, Least Absolute Shrinkage and Selection Operator (LASSO) penalisation has the capacity to fix parameters coefficient to null. The ENET regularisation combines both Ridge and LASSO penalisation^39^. Hence, the multivariate linear model $h^\text {(enet)}: X^* \rightarrow Y$ is

$$\begin{aligned} y_t^\text {(enet)} = \mathbf {x_t}^t \beta + \varepsilon _t \, , \end{aligned}$$

with ${\mathbf {x}} \in X^*$ the predictors, $\beta \in {\mathbb {R}}^{d}$ the parameters of the model and $\epsilon _t$ the error term. The regularisation stems from the optimisation of the objective

$$\begin{aligned} \min _{\beta \in {\mathbb {R}}^d} \quad \frac{1}{2} ||y_t^\text {(enet)} - y_t||_2^2 + \lambda \left( (1-\alpha ) ||\beta ||^2_2 + \alpha ||\beta ||_1 \right) , \end{aligned}$$

where $\alpha \in [0,1]$ denotes the mixing parameter which defines the balance between the Ridge regularisation and the LASSO regularisation. $\lambda$ denotes the impact of the penalty with $\lambda \rightarrow \infty$. For $\alpha = 0$ and $\alpha = 1$, the model will use a ridge and a lasso penalisation, respectively. Thus, for $\alpha \rightarrow 1$ and a fixed value of $\lambda$, the number of removed variables (null coefficients) increases with monotony from 0 to the LASSO most reduced model. The model was adjusted by hyper-parameters $\alpha$ and $\lambda$ during the model selection, being part of the CV process (as described below).

##### Principal component regression

 In this multivariate context with potential multicollinearity issues, principal component analysis aims to project the original data set from $X^*$ into a new space ${\tilde{X}}^*$ of orthogonal dimensions called principal components. These dimensions are built from linear combinations of the initial variables. One may use the principal components to regress the dependent variable: also known as Principal Components Regression (PCR). The regularisation is performed by using as regressors only the first principal components which retain the maximum of variance of the original data, by construction. In our study and according to the Kaiser’s rule^48^, *p* principal components with an eigenvalue higher than 1 were retained and further used in linear regression.

Such a model, $h^{(pcr)} : {\tilde{X}}^* \rightarrow Y$, can be defined as a linear multivariate regression over principal components as

$$\begin{aligned} y^{(pcr)}_t = \tilde{{\mathbf {x}}}^t_t \beta + \varepsilon _t \, , \end{aligned}$$

with ${\mathbf {x}} \in {\tilde{X}}^* \, \backslash \{\tilde{X^*}_{p+1}, \ldots , \tilde{X^*}_{d}\} \,$ the predictors, $\beta \in \mathbb R^{p}$ the parameters of the model and $\epsilon _t$ the error term. In addition to being a regularisation technique by using a subset of principal components only, PCR also exerts a discrete shrinkage effect on the low variance components (the lower eigenvalue components), nullifying their contribution in the original regression model.

#### Random forest

Random Forest model consists of a large number of regression trees that operate as an ensemble. RF is random in two ways, (i) each tree is based on a random subset of observations and (ii) each split within each tree is created based on a random subset of candidate variables. The overall performance of the forest is defined by the average of predictions from the individual trees^49^. In this study, random subset of variables and number of trees were the two hyper-parameters for adjusting the model within the model selection. The model is a function $h^\text {(rf)} : X^* \rightarrow Y$.

### Time series cross-validation and prediction

Since we aim at predicting daily skating performances such as non-independent and identically distributed random variables, the time dependencies have to be accounted for in the cross-validation procedure. It ensures information from the future are not used to predict performances of the past. Hence, data were separated—respectively to the time index—into one training data set for time series CV (80% of the total data) and the remaining data for an unbiased model evaluation (evaluation data set). In this procedure, a model selection occurs first with the search of hyper-parameters values that minimise the predictive model error over validation subsets. The model selection is detailed in Algorithm 1.

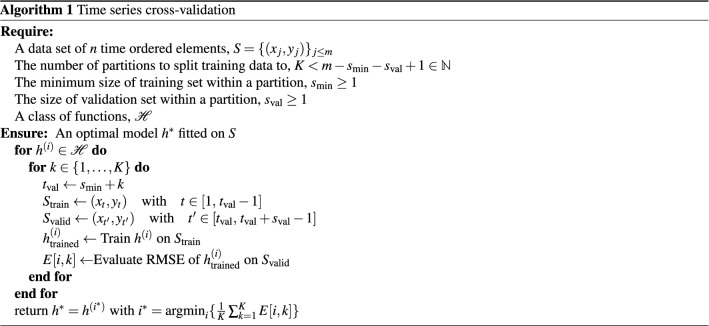



Algorithm 1 iteratively evaluates a class of functions ${\mathscr {H}}$, in which each function $h^{(i)}$ differs from its hyper-parameters values. A time ordered data set *S* is partitioned into training and validation subsets ($S_{train}$ and $S_{valid}$, respectively). For each partition *k* with $k \in \{1,...,K\}$, $h^{(i)}$ functions are fitted on the incremental $S_{train}$ and evaluated on the fixed $S_{valid}$ subset that occurs after the last element of $S_{train}$. Once $h^{(i)}$ functions are evaluated on *K* partitions of *S*, a function $h^{(i^*)}$ that provides the lowest and averaged root mean square error (*RMSE*) among validation subsets defines an optimal model denoted $h^*$.

#### Model evaluation

Afterwards and for each partition of *S*, $h^*$ is adjusted on new time ordered training subsets $S'_{train}$ which combines both $S_{train}$ and $S_{valid}$. Then, the generalisation capability of $h^*$ is evaluated on fixed length subsets of evaluation data $S_{eval}$, saved for that purpose. This procedure refers to the so-called “evaluation on a rolling forecasting origin” since the “origin” at which the forecast is based rolls forward in time^50^. Note that the DR is only concerned by the model evaluation step since it has no hyper-parameters to be optimised in the model selection phase.

### Statistical analysis

For any model, the goodness of fit according to linear relationships and to performance were described by the coefficient of determination ($R^2$) and the *RMSE* criterion respectively. Their generalisation ability is described by the difference between *RMSE* computed on each training and evaluation data. The prediction error was reported through the Mean Absolute Error (*MAE*) between observations and predictions. After checking normality and variance homogeneity of the dependant variable by a Shapiro-wilk and a Levene test respectively, linear mixed models were performed to assess the contribution of each class of model over the modelling error rate. Inter and intra subject variability over athletic performances modelling have been considered through random effects. Repeated measure ANOVAs were performed in order to assess the effect of the model class and population over the response, the effect size being reported through $\eta ^{2}$ statistic. Multiple pairwise comparison of errors between the model of reference and the other models were performed using Dunnett’s post-hoc analysis. Significance threshold was fixed to $p < 0.05$. For linear mixed models, unstandardised regression coefficients $\beta$ are reported along with 95% confidence interval (*CI*) as a measure of effect size. Models computation and statistical analysis were conducted with R statistical software (version 4.0.2). The DR model was computed with personal custom-built R package (version 1.0)^51^.

## Results

**Figure 2:**
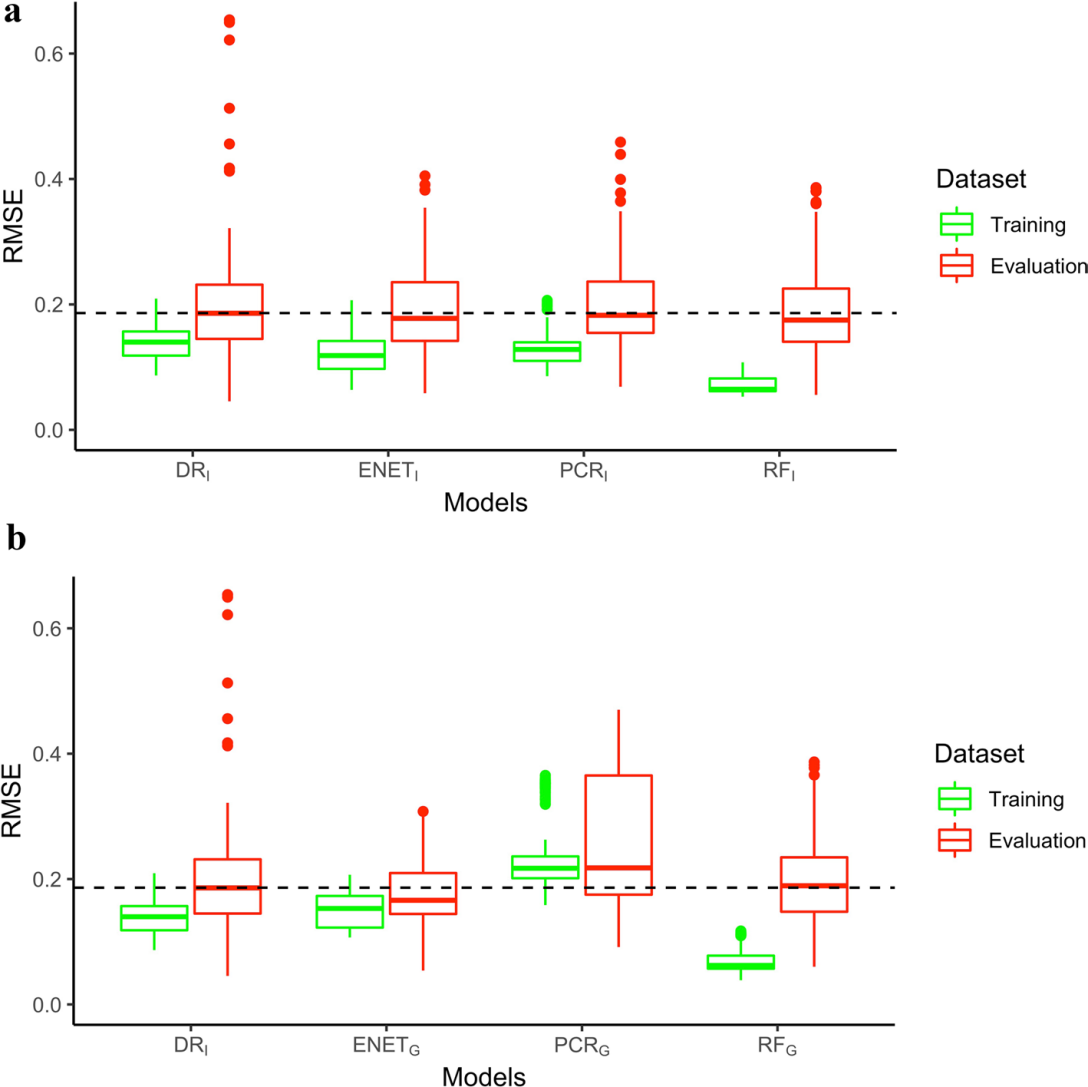
Distributions of models’ performance. (**a**) RMSE distributions of each individual models and (**b**) the models computed on the whole group. Within boxplot midline represents the median of the distribution. All of them are compared to the dose-response (DR) model.

Through the times series CV, models provided heterogeneous generalisation and performance prediction. Distributions of RMSE per model are illustrated in Fig. 2.

### Models generalisation

Mixed model analysis showed that both $\text {ENET}$ and $\text {PCR}$ models lowered the differences in terms of prediction errors between the training and evaluation data set ($\beta = -0.023 \in [-0.037, -0.007] \, 95\% \, CI$, $p = 0.003$ and $\beta = -0.057 \in [-0.065, -0.047] \, 95\% \, CI$, $p < 0.001$ for $\text {ENET}_I$ and $\text {ENET}_G$; $\beta = -0.026 \in [-0.040, -0.011] \, 95\% \, CI, \, p < 0.001$ and $\beta = -0.032 \in [-0.041, -0.022] \, 95\% \, CI$, $p < 0.001$ for $\text {PCR}_I$ and $\text {PCR}_G$, respectively). A significant effect of the model class on the generalisation risk was also reported ($p < 0.001, \, \eta ^2 = 0.23 \in [0.20, 0.26]\, 95\% \, CI$). The most generalisable models were ENET and PCR models computed on overall data, followed by individual based models. Generally, group-built models likely provided a greater generalisation capability than individual based models ($\beta _{diff} = -0.0144, \, p < 0.001, \, \eta ^2 = 0.01 \in [0.00, 0.01]\, 95\% \, CI$). A summary of model pairwise comparisons is provided in Table 2.

**Table 2:** Summary of models pairwise comparisons for generalisation and prediction abilities.

Comparison	$\beta _{diff}$	*t* ratio	*p*	Criterion
$\text {DR}_I - \text {ENET}_G$	0.057	− 11.841	< 0.001	Generalisation
$\text {DR}_I - \text {PCR}_G$	0.032	6.644	< 0.001	Generalisation
$\text {DR}_I - \text {PCR}_I$	0.026	3.365	0.004	Generalisation
$\text {DR}_I - \text {ENET}_I$	0.023	2.933	0.018	Generalisation
$\text {DR}_I - \text {RF}_G$	− 0.027	− 5.649	< 0.001	Generalisation
$\text {DR}_I - \text {RF}_I$	− 0.028	− 3.831	< 0.001	Generalisation
$\text {DR}_I - \text {ENET}_G$	0.041	5.607	< 0.001	Prediction
$\text {DR}_I - \text {RF}_G$	0.022	3.067	0.012	Prediction
$\text {DR}_I - \text {ENET}_I$	0.021	2.112	0.156	Prediction
$\text {DR}_I - \text {RF}_I$	0.018	1.789	0.294	Prediction
$\text {DR}_I - \text {PCR}_I$	0.016	1.537	0.438	Prediction
$\text {DR}_I - \text {PCR}_G$	− 0.042	− 5.779	< 0.001	Prediction

$\beta _{diff}$ represents the marginal mean difference of the RMSE distribution between the DR model and its comparison.

### Prediction performances

Root mean square errors reported on evaluation data using mixed model analysis indicated that $\text {ENET}_G$ was the most contributing model in lowering the prediction errors ($\beta = -0.041 \in [-0.055, -0.027] \, 95\% \, CI, \, p < 0.001$), followed by $\text {RF}_G$ as shown in Table 2. Accordingly, a significant model class effect on prediction errors was reported ($p < 0.001, \, \eta ^2 = 0.18 \in [0.15, 0.21]\, 95\% \, CI$). Computing models over a larger population (i.e. group-based models) showed only a trend in favour of group-based models over the errors response rate ($p = 0.146$).

Distributions of *RMSE* on data used for model evaluation have shown heterogeneous variance between models. The greatest standard deviations were found for $\text {DR}_I$ and $\text {PCR}_G$ with $\sigma = 0.053$ and $\sigma = 0.062$ respectively. The ENET, $\text {PCR}_I$ and RF models provided more consistent performances with lower standard deviations comprised within [0.023; 0.027] and [0.012; 0.017] intervals for individual and group computed models, respectively. Note that the greatest errors on evaluation data were systematically attributed to one particular athlete. In average, predictions made from this athlete led to greater RMSE than ones made from other athletes ($p < 0.001$, $\beta _{diff} = 0.22 \, [0.163, 0.286] \, 95\% \, CI$). Mean values of $R^2$ indicated that weak linear relationships between performance and predictors were identified by models ($R^2 \in [0.150 ; 0.206]$). The highest averaged $R^2$ value but also the greatest standard deviations were reported for $\text {DR}_I$ models ($R^2 = 0.206 \pm 0.093$). However, significant differences of averaged $R^2$ were only found for $\text {ENET}_I$, $\text {RF}_G$ and $\text {PCR}_G$ ($\beta = -0.056 \; [-0.10; -0.01] \; 95\% \; CI$, $p =0.02$; $\beta = -0.041 \; [-0.08; -0.01] \; 95\% \; CI$, $p = 0.02$ and $\beta = -0.036 \; [-0.07; -0.01] \; 95\% \; CI$, $p = 0.04$ respectively). A summary of model performances is provided in Table 3.

**Figure 3:**
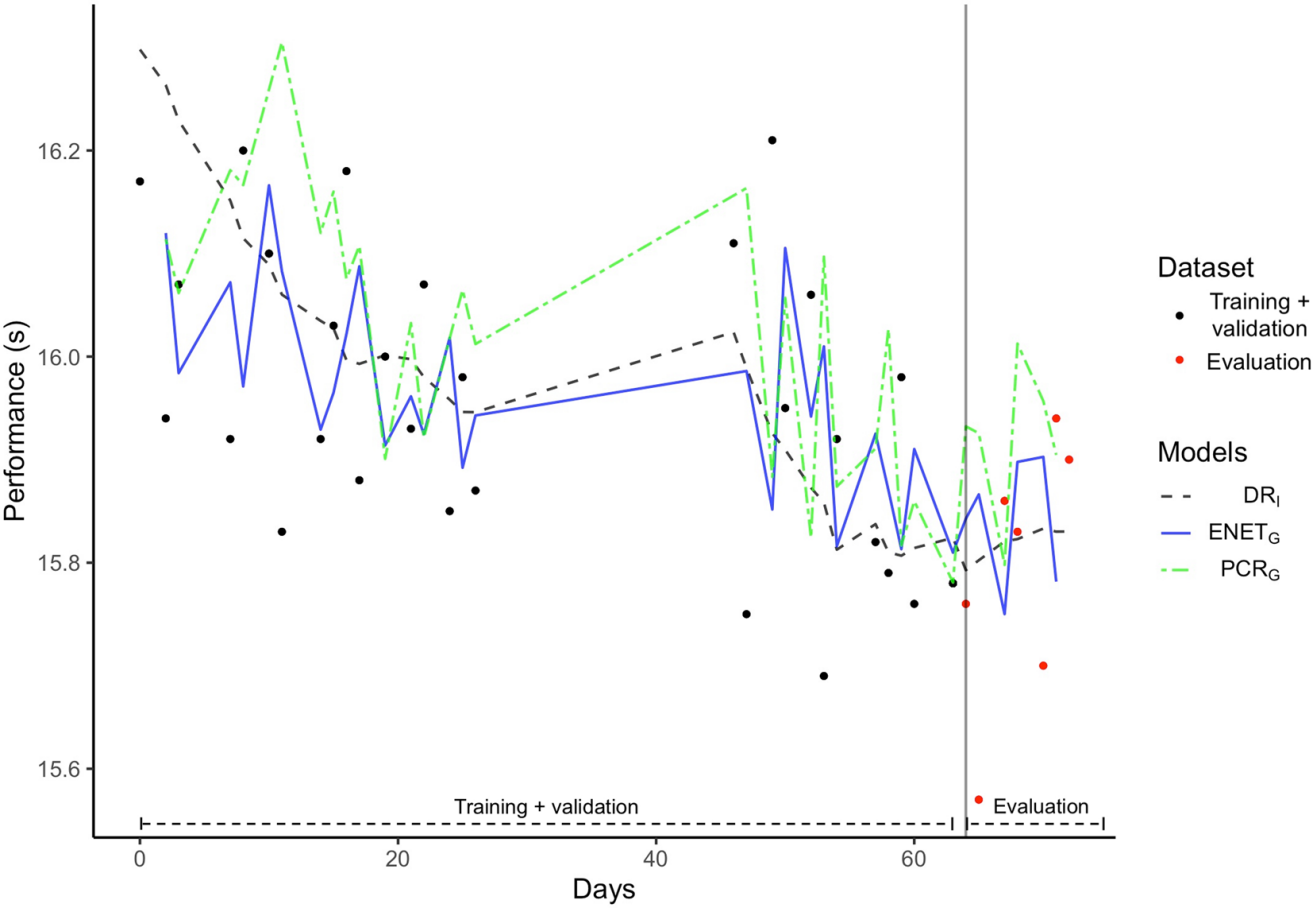
Modelled performance of a representative subject. Solid and dashed lines represent the DR model and the two models offering the best generalisation. On this example, the training data set (80% of the data that combines training and validation subsets) and evaluation data set (20% of the data, the evaluation subset) areas are separated by the vertical solid line. Fitted parameters of the DR model were $k_1 = -2.45e{-}05$, $k3 = - 2.58e{-}09$, $\tau _1 = 39$, $\tau _2 = 26$, $\tau _3 = 5$. Hyper-parameters of the PCR and ENET models were $n \; comp = 3$ and $\alpha = 0.28$, $\lambda = 0.02.$

Predictions made from the two most generalisable models—$\text {ENET}_G$ and $\text {PCR}_G$—and the reference $\text {DR}_I$ illustrate the sensitivity of models for a representative athlete (Fig. 3). Performances modelled from $\text {DR}_I$ model were relatively steady and less sensitive to real performance variations. Standard deviation calculated on data used for model evaluation supported such a smooth prediction with $\sigma = 0.015$, $\sigma = 0.071$ and $\sigma = 0.062$ for $DR_i$, $PCR_G$ and $ENET_G$, respectively. Regarding $ENET_G$, the greatest standardised coefficients were attributed to the auto-regressive component (i.e. the past performance) such as $\beta = 0.469$, followed by the athlete factor and then impulse and serial bi-exponential aggregations. For regression, $PCR_G$ used the three first principal components explaining 52.3%, 16.5% and 7.6% of the total variance, respectively. Details about models’ parameters as well as principal component compositions are available on Supplementary material Appendix 2.

**Table 3:** Summary of the predictive models.

Model	$R^2$	MAE	RMSE	Hyper parameters*
$\text {DR}_I^*$	**0.206 ± 0.093**	0.189 ± 0.055	0.225 ± 0.053	$\overline{k_1} = - 3.95e{-}05, k_1 \in [- 4.85e{-}05; - 3.19e{-}05]$
				$$\overline{k_3} = - 7.75e{-}09, k_3 \in [- 4.01e{-}09; - 1.71e{-}08]$$
				$\overline{\tau _1} = 36.02, \tau _1 \in [25.82 ; 42.28]$, $\overline{\tau _2} = 22.57, \tau _2 \in [14.58 ; 26]$, $\overline{\tau _3} = 5.23, \tau _3 \in [4.33 ; 6.67]$
$\text {ENET}_I$	0.150 ± 0.010	0.169 ± 0.020	0.197 ± 0.023	${\overline{\alpha }} = 0.176, \alpha \in [0;0.6]$, ${\overline{\lambda }} = 0.273, \lambda \in [0;1]$
$\text {PCR}_I$	0.164 ± 0.068	0.173 ± 0.025	0.201 ± 0.027	$\overline{n \, comp} = 1.918, n \, comp \in [1;3]$
$\text {RF}_I$	0.193 ± 0.074	0.170 ± 0.023	0.199 ± 0.024	$\overline{m try} = 8.90, m try \in [1;17]$
$\text {ENET}_G$	0.179 ± 0.063	**0.150 ± 0.010**	**0.176 ± 0.012**	$\alpha = 0.28, \lambda = 0.02$
$\text {PCR}_G$	0.17 ± 0.053	0.22 ± 0.044	0.259 ± 0.062	$n comp = 3$
$\text {RF}_G$	0.164 ± 0.069	0.163 ± 0.017	0.195 ± 0.017	$m try = 16$

According to model families, criteria were averaged among folders and displayed with their standard deviation. For individual models, averaged values of hyper parameters are displayed along with lower and upper recorded values. The greatest performance among criteria is listed in bold type.

*Indicates the $\text {DR}_I$ as the reference model and specification of its averaged parameters.

### Discussion

In the present study, we provided a modelling methodology that encompasses data aggregation relying on physiological assumptions and model validation for future predictions. Data were obtained from elite athletes, able of improving their performance by training and being very sensitive to physical, psychological and emotional states. The variable dose-response model^13^ was fitted on individual data. It was compared to statistical and machine-learning models fitted on individual and on overall data: ENET, PCR and RF models.

Cross validation outcomes revealed significant heterogeneity in performances of models, even though the differences remain small regarding the total time of skating trials (see Table 3). The main criterion of interest, generalisation, was significantly greater for both ENET and PCR models than $\text {DR}_I$ model. One can explain this result by the capabilities of the statistical models to better catch the underlying skating performance process using up to 19 independent variables when associated with regularisation methods. Conversely, the $\text {DR}_I$ model relies on two antagonistic components strictly based on the training load dynamics. It does not deal with any other factors that may greatly impact the performance (e.g. psychological, nutritional, environmental, training-specific factors)^12,18,52^. Thus, such a conceptual limit can be overtaken by employing multivariate modelling that may result in a greater comprehension of the training load - performance relationship, for the purpose of future predictions^9,12^. To date, only a recent study from Piatrikova et al.^53^ extended the former Fitness–Fatigue model framework^3^ to account for some psychometric variables as model inputs. Despite the authors reported an improved goodness of fit for this multivariate alternative, attributing impulse responses to these variables might question the conceptual framework behind the model.

Distributions of RMSE from training and evaluation data sets allow us to establish a generalisation model ranking (Table 2). Linear models computed on overall data offer a better generalisation. This finding is essential because by handling the bias-variance trade-off, models are more suited for capturing a proper underlying function that maps inputs to the target even on unknown data. Hence, it allows further physiological and practical interpretations from the models such as the remodelling process of skeletal muscle involved by exercise, dynamically represented by exponential growth and decay functions^28^. Besides, this result might be partly explained by the sample size. It is well known that statistical inference on small samples leads to bad estimates and consequently to bad performances in prediction^54,55^. A greater sample size obtained by combining individual data led to more accurate parameter estimates, being more suitable for sport performance modelling^12^. That is particularly important to consider when we aim to predict a very few discipline specific performances throughout a season. However, predicting non-invasive physical quality assessments which can be daily performed (e.g. squat jumps and its variations for an indirect assessment of neuromuscular readiness^56^, short sprints) may be an alternative for small sample size issues. In our case, standing start time trials over 1.5 laps allowed for the coach to evaluate underlying physical abilities of the skating performance, several times a week. Also, regularisation tends to stabilise parameters estimators and favour generalisation of the models. For instance, multicollinearity may occur in high-dimensional problems and stochastic models generally suffer from such a conditioning. One would note that the ENET and PCR models attempt to overcome these issues in their own way by (i) penalising or removing features—or both—that are mostly linearly correlated and (ii) by projecting the initial data space onto a reduced space, which is optimised to keep the maximum of variance of the data from linear combinations of the initial features. Both approaches limit the number of unnecessary—or noisy—dimensions. In contrast, in this study non-linear machine learning models ($RF_I$ and $RF_G$) expressed a lower generalisation capability than linear models even when models combine data from several athletes. We believe that such models may be powerful in multidimensional modelling but require an adequate data set with, in particular, ones with a sufficient sample size. Otherwise, model overfitting may occur at the expense of inaccurate predictions on unknown data.

As reported previously and with the exception of $\text {PCR}_G$, models were more accurate in prediction than $\text {DR}_I$ (Table 3). The large averaged RMSE as well as large standard deviations provided by the $\text {DR}_I$ among performance criteria tend to agree with the literature, since the model is prone to suffer from a weak stability and ill-conditioning raised by noisy data that impact its predictive accuracy^9,10^. This evokes that linear relationships between the two components “Aptitude”—“Fatigue” and the performance are not clear. However, because of a lack of cross-validation procedures on impulse response models and particularly the DR employed in our study, our results cannot be validly compared with the literature. Despite lower standard deviations of $R^2$ reported by ENET and PCR models, the weak averaged $R^2$ values suggest that linear models can only explain a few part of the total variance. Note that all linear models are concerned (including the $DR_I$), since the differences in averaged $R^2$ between models are relatively small and only significant for $ENET_I$, $RF_G$ and $PCR_G$ models. Therefore and if the data allow it (i.e. a sufficient sample size and robustly collected data), non-linear models may still be effective and should be considered during the modelling process.

The sensitivity of models according to gains and losses of performances differed between the two most generalisable models—$\text {ENET}_G$ and $\text {PCR}_G$—and the reference $\text {DR}_I$ (Fig. 3). Such differences can be explained by the influence of variables that may affect performance, other than training loads dynamic (e.g. ice quality the day of performance, cumulative training loads following a serial and bi-exponential function, the last known performance) or a $\text {DR}_I$ model failure in parameter estimates regarding to the variability of the data. Indeed, parameters estimates of $ENET_G$ supported that since changes in skating performance were mostly explained through the past performance, weighted by individual properties and to a lesser degree by training related parameters. The $PCR_G$ used a different approach for the same purpose and greatly relied on training related aggregations as well as environmental and training programming variables (see Appendix 2). However, this applied example does not inform us about neither the generalisation ability of models nor accuracy of predictions because it concerns only a particular set of data, where the selected models (i.e. with optimal hyper-parameters) are trained on the first 80% of data and evaluated on the 20% remaining data. In addition, since model estimates greatly depend on the sample size, we might expect significant different estimates with more data (particularly for $ENET_G$).

This study presents some limits. The first one concerns the data we used and particularly the criterion of performance: standing start time trials few times a week during an approximately 3-months period. Even though being a very discipline specific test in which athletes are familiar and being conducted in standardised conditions, each test requires high levels of arousal, buy-in, motivation and technique. Therefore, psychological states and cognitive functions monitoring such as motivation and attentional focus^57,58^ should have been done prior performing each trial. A concrete example is provided through the Fig. 3, where $ENET_G$ greatly penalised the training correlated features and kept the influence of the auto-regressive component predominant over other features. This may be the consequence of either an inference issue due to the relative small sample size, or a lack of informative value of training related features that do not allow to explain changes in skating performance. Also, both reasons support models failure in predicting skating performances of one particular athlete, who showed significant greater errors of prediction. It emphasises the importance of measuring the “right” variables for performance modelling purposes, in particular if the sport-specific performance involves various determining factors.

Secondly, the time series cross-validation presented here has a certain cost, most notably when only few data are available (e.g. when models are individually computed). The rolling origin re-calibration evaluation performed as described by Beirgmer et al.^59^ implies a model training only on a incremental sub-sequence of training data. Hence, the downsized sample size of the first training sub-sequences may cause model failure in parameter estimates and consequently, an increase of prediction errors. Then, training and evaluation data sets present some dependencies. In order to evaluate models on fully independent data, some modifications of the current CV framework exist at the expense of withdrawing even more data in the learning procedure. According to Racine^60^, the so-called *hv - block cross-validation* is one of the least costly alternative to the CV used in our study, requiring a certain gap between each training and validation subsets. However, due to a limited sample size, we voluntary chose to not adapt the original CV framework described in Algorithm 1. Nonetheless, we recommend researchers and practitioners to consider such alternatives in case of significant dependencies and when sample size is sufficient.

Finally, *backtesting* was performed in order to evaluate model performances on historical data. From a practical point of view, models are able to predict the coming performance following a given feature of data known until day *t*. However, the contribution of training load responses modelling also concerns training after-effects simulations over a longer time frame. Having identified a suitable model, practitioners may pinpoint key performance indicators—specific to the discipline of interest—and confront model estimates to field observations. Then, simulations of these independent variables within their own distributions would allow practitioners and coaches to simulate changes in performance following objective and subjective measures of training loads, and any performance factors that are monitored. Conditional simulations that consider known relationships between independent variables (e.g. relationships between training load parameters)^61,62^ may improve the credibility of simulations.

The modelling process presented so far constitutes a part of a decision support system (DSS), from issue and data understanding to evaluation of the modelling results^63^. Supported by a deployment framework that makes models usable by all, DSS helps technical, medical staffs in the training programming and scheduling tasks^64^ throughout a systemic and holistic approach of a complex problem, such as athletic performance^65^. Besides, the technological improvement of sports wearable sensors and underpinning available data for quantifying and characterising exercise foster the development of DSS in individual and team sports.

## Conclusion

In this study, we provided a transferable modelling methodology which relies on the evaluation of models generalisation ability in a context of sport performance modelling. The mathematical variable dose-response model along Elastic net, principal component regression and random forest models were cross-validated within a time series framework. Generalisation of the DR model was outperformed by ENET and PCR models, though our results may not be directly compared with the literature. The ENET model provided the greatest performances both in terms of generalisation and accuracy in prediction when compared to the DR, PCR and RF models. Globally, increasing sample size by computing models on the whole group of athletes led to more performing models than the individually computed ones. Yet, our results should be interpreted in the light of the models used. In our study, we foster the use of regularisation and dimension reduction methods for addressing high dimensionality and multicollinearity issues. However, other models could stand valuable for athletic performance modelling (e.g. mixed-effect models for repeated measures, generalised estimating equations since there are possible unknown correlation between outcomes, autocorrelation and cross-correlation functions for time-series analysis).

The methodology highlighted in our study can be reemployed whatever the data, with the aim of optimising elite sport performance through training protocols simulations. Beyond that, we believe that model validation is a requisite for any physiological and practical interpretation for the purpose of making future predictions. Further researches that involve training session simulations and model evaluations in forecasting would highlight the relevance of some model families for training programming optimisation.

## Supplementary Information

Supplementary Information.
