# Supplementary material for: Training load responses modelling and model generalisation in elite sports
Source: Sci Rep. 2022 Jan 28;12:1586. doi: 10.1038/s41598-022-05392-8 (PMC8799698; doi:10.1038/s41598-022-05392-8)
Supplement: Supplementary file 1 — Supplementary Information. [file 41598_2022_5392_MOESM1_ESM.pdf]

Training load responses modelling and model generalisation in elite sports

Frank Imbach[1,2,3,*], **Stephane Perrey**[2], **Romain Chailan**[1], **Thibaut Meline**[3,4], **and Robin Candau**[3]

[1]Seenovate, Montpellier, France
[2]EuroMov Digital Health in Motion, Univ Montpellier, IMT Mines Ales, Montpellier, France
[3]DMeM, Univ Montpellier, INRAe, Montpellier, France
[4]Fédération Française des Sports de Glace, Paris, France
[*]frank.imbach@umontpellier.fr

Appendix 1

Specific training

On-ice sessions refer to specific training. Session TLs were calculated from Power Output (PO, W), volume and ice properties. Individual PO depends on power required to change kinetic energy (P_{kin}), power required to overcome air and ice resistance (P_{aero} and P_{ice} respectively). Let us define

$$
\begin{aligned}
P_{kin} &= \frac{\frac{1}{2}(mv_f^2 - v_i^2)}{t},\\
P_{aer} &= \frac{1}{2} AC_D\, \rho\, v^3 \quad \text{and}\\
P_{ice} &= C_f\, m\, g\, \bar{v}.
\end{aligned}
$$

In this context, m denotes the mass of the athlete and that of the equipment, v_f is the maximal velocity reached during the run, v_i is the initial velocity being null, $\bar{v}$ is the mean velocity and t is the exercise duration. The effective frontal area AC_D is a standardised fixed value of $0.25\,\mathrm{m}^2$ according to subjects corpulence and[1]. Also, ρ denotes the air density recorded at 1850 meters above sea and is equal to $1.029\,\mathrm{kg.m}^{-3}$. The friction coefficient C_f is standardised as $C_f = 0.006$, according to maximal values found by[2] and due to a track with sharper turns. Finally, g denotes the acceleration due to the gravity, equal to $9.80665\,\mathrm{m.s}^{-2}$.
Thus,

$$PO = P_{kin} + P_{aer} + P_{ice}.$$

Relative intensity of the session (I_{ice}, as a percentage of the maximal PO) can now be determined as

$$I_{ice} = \frac{I_{ice}^f N_f + I_{ice}^b (N - N_f)}{N}. \tag{S1}$$

This relative exercise intensity includes both forward and backward positions denoted I_{ice}^f and I_{ice}^b respectively, with

$$
\begin{aligned}
I_{ice}^f &= \frac{PO}{\max PO} + C,\\
I_{ice}^b &= I_{ice}^f - E\, I_{ice}^f.
\end{aligned}
$$

Here, C denotes the ice impact on skating for an ice quality (Q_{ice}) arbitrary measured by athletes on a 0-10 Borg scale and averaged. If Q_{ice} is below 7.5 arbitrary units (a.u), a linear penalisation is attributed such as $C = -0.008\, Q_{ice} + 0.06$, where α

and β coefficients were estimated from at least two equal performances with different values of Q_{ice}. In addition, E denotes the skating economy due to drafting and N denotes the overall number of laps with also a distinction for the forward position (N_f). Finally, ice session training load is

$$TL_{ice} = I_{ice}\, V\, K\, (\frac{I_{RPE}}{\max I_{RPE}})\, \rho, \tag{S2}$$

where V is the volume parameter defined as the product of the number of laps run and the distance of a lap; K depends on the subject's gender with $K = 0.64\, e^{1.92\, I}$ for males and $K = 0.86\, e^{1.67\, I}$ for females respectively and according to[3]; I_{RPE} is the rate of perceived exertion quoted on a 6-20 Borg scale, $\max I_{RPE}$ is the maximal value that can be quoted ($\max I_{RPE} = 20$); ρ denotes the density parameter, such as $\rho = \frac{1}{2}\rho_s$ with ρ_s the density of the session (%) which represents the effective work done by the athlete.

Non-specific training

Training loads of resistance training (TL_{RT}), aerobic training (TL_{aer}), repeated sprint training (TL_{RS}) and activation sessions (TL_{act}, specific warm-up) were also quantified as

$$TL_{RT} = I_{RT}\, V\, K\, (\frac{I_{RPE}}{max\, I_{RPE}})\, \rho, \tag{S3}$$

$$TL_{aer} = I_{RPE}\, T\, K\, \rho_s\, k_{aer}, \tag{S4}$$

$$TL_{RS} = I_{RS}\, V\, K\, (\frac{I_{RPE}}{max\, I_{RPE}})\, \rho \quad \text{and} \tag{S5}$$

$$TL_{act} = I_{RPE}\, T\, K\, \rho_s\, k_{off}. \tag{S6}$$

Here I_{RT} denotes the intensity in percentage of the maximal repetition, V is the volume defined by the number of repetitions, T is the total time of exertion, k_{aer} and k_{off} denote a weighting factor for aerobic and activation exercises such as $k_{aer} = 5$ a.u (empirically defined by the coach) and $k_{off} = 15$ a.u respectively. Any of the training sessions are weighted by I_{RPE}. However, a specific intensity was only quantifiable for TL_{RT} and TL_{RS} and further considered in the training load calculation. According to the training condition, Equations S2 – S6 respectively define the discrete function $w(t)$.

Appendix 2

Details of models used in Figure 3.

Elastic net regularisation

Parameter	Coefficient
Intercept	16.84
past_perf	0.47
S4	0.11
S6	0.06
ser_density	0.02
imp_load	0.01
imp_density	0.01
imp_relativeInt	0.00
imp_avgPower	0.00
imp_RPE	0.00
imp_session_duration	0.00
ser_load	0.00
ser_avgPower	0.00
ser_maxPower	0.00
ser_RPE	0.00
ser_relativeInt	0.00
ser_session_duration	0.00
ice_quality	-0.00
rest_days	-0.01
imp_maxPower	-0.03
S7	-0.15
S3	-0.18
S2	-0.22
S5	-0.23

Principal component regression: contribution of variables per dimensions

Independent variable	contribution (%)	Dimension
ser_load	10.07	1
imp_relativeInt	9.99	1
ser_RPE	9.75	1
ser_relativeInt	9.70	1
ser_maxPower	9.33	1
imp_density	28.21	2
imp_session_duration	22.20	2
imp_RPE	14.24	2
ser_density	10.01	2
ser_session_duration	4.61	2
rest_days	28.70	3
ser_density	14.68	3
ice_quality	11.97	3
imp_load	11.19	3
imp_RPE	10.20	3

References

1. Van Ingen Schenau, G. J. The influence of air friction in speed skating. *J. Biomech.* **15**, 449–458 (1982).

2. De Koning, J. J., De Groot, G. & van Ingen Schenau, G. J. Ice friction during speed skating. *J. Biomech.* **25**, 565–571 (1992).

3. Banister, E. & Calvert, T. Planning for future performance: implications for long term training. *Can. J. Appl. Sport Sci.* **5**, 170–176 (1980).
